# Multifactorial Optimization of Gluten‐Free Cookie With Artichoke Bracts as Rice Flour Substitute and Transglutaminase

**DOI:** 10.1002/fsn3.70420

**Published:** 2025-06-10

**Authors:** Ceyda Dadalı, Yağmur Özcan, İrem Cansu Ensari

**Affiliations:** ^1^ Engineering Faculty, Department of Food Engineering Ege University İzmir Türkiye

**Keywords:** artichoke bracts, *Cynara scolymus* L., gluten‐free cookie, optimization, transglutaminase

## Abstract

The disposal of food waste represents a significant concern within the food industry. Artichoke waste accounts for over 80% of the total mass of artichokes, and artichoke bracts are also a significant component of this waste. This study aimed to utilize artichoke bracts as a substitute for rice flour in gluten‐free cookies, with a focus on optimizing the usage ratio of rice flour substitute and transglutaminase enzyme. Rice flour substitute, designated as an independent variable, was configured within the range of 0%–30%, while transglutaminase was set at 0%–1% using a Face Centered Central Composite Design. The optimization process resulted in gluten‐free cookies containing 18.65% artichoke bracts as a rice flour substitute and 0.99% transglutaminase enzyme. The study established that the optimum gluten‐free cookie exhibited higher antioxidant activity (2.89 μmol TE/g) and total phenolic content (0.74 mg GAE/g) and could be categorized as a high fiber content food (7.97 g/100 g), in comparison to the control gluten‐free cookie (*p* < 0.05). Furthermore, an increase was observed in the hardness (23.38 N) and fracturability (42.21 mm) values of the optimum gluten‐free cookie (*p* < 0.05). While L* and b* values from the color properties were lower than the control gluten‐free cookie, the a* value was higher (*p* < 0.05). The artichoke bracts and transglutaminase enzyme were effective in the development of the sensory properties (color, texture, flavor, and overall acceptability) of gluten‐free cookies. This study demonstrated that artichoke bracts and transglutaminase can enhance the quality of gluten‐free cookies while concurrently promoting sustainable food production and generating a value‐added product by utilizing artichoke bracts as a substitute for rice flour.

## Graphical abstract

This study aimed to utilize artichoke bracts as a substitute for rice flour in gluten‐free cookies, with a focus on optimizing the usage ratio of rice flour substitute and the transglutaminase enzyme. Rice flour substitute, designated as an independent variable, was configured within the range of 0%–30%, while transglutaminase was set at 0%–1% using a Face Centered Central Composite Design. The optimization process resulted in gluten‐free cookies containing 18.65% artichoke bracts as a rice flour substitute and 0.99% transglutaminase enzyme.

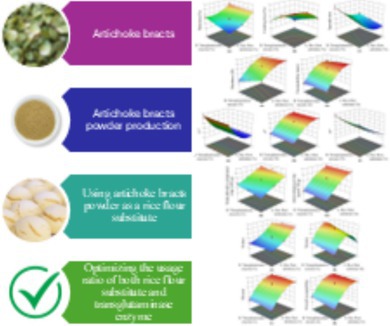

## 1 Introduction

In recent years, there has been an increase in the demand for gluten‐free nutrition due to the spread of diseases such as celiac disease, gluten intolerance, wheat allergy, gluten ataxia, and the resulting awareness of these diseases (Proetto et al. 2024). Celiac disease affects approximately 1% of the world's population and occurs when the villi in the small intestine are destroyed, reduced, and shrunk due to the consumption of gluten. Gluten is found in wheat, barley, rye, and their derivatives, resulting in malnutrition due to the inability to absorb consumed food (Gulsunoglu‐Konuskan et al. 2024). The only treatment for celiac disease is to consume gluten‐free food products and follow a gluten‐free diet for life. Moreover, even in the absence of celiac disease or gluten sensitivity, there is a growing number of individuals who prefer gluten‐free products. Consequently, the gluten‐free product market is undergoing continuous development in response to the rising demand for gluten‐free products (Jeong et al. 2021; Gulsunoglu‐Konuskan et al. 2024).

Gluten‐free food products generally have a limited product variety. Among these products, gluten‐free bakery products have a very important place in the gluten‐free diet. Gluten‐free foods can be produced by using gluten‐free grain flours such as corn, buckwheat, rice, chickpea, soy, etc. These grain flours have high starch content but low protein, fiber, vitamins, minerals, and phenolic compounds (Tunçer and Ayhan 2021). In addition, gluten affects the volume, elasticity, and crust structure of the product by ensuring the retention of gases formed during the fermentation process in dough formation. Therefore, it is an important protein to produce cereal products such as bread, cake, cookies, pasta, and noodles (Ceylan and Muştu 2021). Gluten‐free products produced using various starch‐rich gluten‐free flours are characterized by low nutritional content and sensory quality deficiencies compared to gluten‐containing alternatives.

The nutritional quality of gluten‐free foods can be increased by using fruit and vegetable wastes having high dietary fiber, protein, antioxidant, and vitamin content (Majzoobi et al. 2016). In recent years, valorization of food, agricultural, and industrial waste and byproducts has become an important issue in order to reduce food waste. The Sustainable Development Goals, which are scheduled to conclude in 2030, were initiated by the United Nations to draw attention to the global issue of food waste (Bartezzaghi et al. 2022). In order to achieve this objective, it is essential to utilize sustainable natural resources in the production of high‐value‐added products (Soares‐Mateus et al. 2023).

Fruits and vegetables are the most wasted food group in food processing and consumption. In addition, the wasted by‐products of fruits and vegetables constitute a potential source, especially in terms of bioactive compounds (Soares‐Mateus et al. 2023). Artichoke ( *Cynara scolymus* L.) is among the vegetables that generate substantial amounts of waste during food processing and consumption. Generally, only the head of the artichoke is consumed, while the bracts and stems, which constitute approximately 80% of the total plant biomass, are discarded as waste (Dadalı 2023a). Approximately 1.59 million tons of artichoke were produced in the world in 2022 (FAOSTAT 2024). The inedible parts of the artichoke constitute a substantial portion of the total biomass, resulting in significant waste generation. These wastes contribute to environmental pollution by causing microbial growth not only in terms of cost but also due to their high moisture content. Artichoke bracts are rich in phenolic compounds, dietary fiber, vitamins, and minerals. Thanks to the bioactive compounds of artichoke bracts, they have many health benefits such as antioxidant, anti‐inflammatory, anticancer, antibacterial, antidiabetic, and antiobesity properties (Bavaro et al. 2025). Artichoke byproducts possess valuable nutritional components and health‐promoting properties. Therefore, they can be used to enrich foods with low nutritional value, especially gluten‐free bakery products (Colombo et al. 2024).

The application of transglutaminase enzymes has been indicated to enhance and develop the textural properties of gluten‐free products. Transglutaminase has been demonstrated to be an effective additive in gluten‐free products, as it enhances protein structure through enzymatic cross‐linking (Saeidi et al. 2018). The addition of transglutaminase to gluten‐free food formulations enhances the viscoelastic properties of dough, thereby improving the quality of products such as bread and cookies by supporting the protein network (Yıldırım 2025).

Artichoke bracts were used in spreadable cheeses (Lucera et al. 2018); biscuits (Eman et al. 2018); bread (Canale et al. 2022, 2023; Bavaro et al. 2025); cake (Dadalı 2023a), fermented cereal soup (Dadalı 2023b); breadsticks (Cannas et al. 2024); gluten‐free bread (Proetto et al. 2024) and gluten‐free cake (Gulsunoglu‐Konuskan et al. 2024). To the best of our knowledge, artichoke bracts have not been used in gluten‐free cookie as a rice flour substitute, and optimization has not been implemented considering rice flour replacer and transglutaminase ratio. The aim of this study is to determine the optimum usage ratio of artichoke bracts as a rice flour substitute and transglutaminase in gluten‐free cookie and to produce a gluten‐free cookie having improved nutritional, physical, and sensory properties.

## 2 Material

Rice flour, sugar, margarine, skimmed milk powder, baking powder, and salt used for gluten‐free cookie production were obtained from local markets in İzmir, Türkiye. Artichoke ( *Cynara scolymus* L.) bracts were purchased from local producers in İzmir. Transglutaminase enzyme was supplied by Smart Chemistry Ind. (Türkiye), and other chemicals used in the analysis were purchased from Merck (Germany).

## 3 Methods

### 3.1 Experimental Design

Response surface methodology was used for the optimization of artichoke bracts and transglutaminase ratio involved in gluten‐free cookie production. A face‐centered central composite design (FCCCD) was utilized to determine the effect of two independent variables on responses and to find the optimum variables. Thirteen experiments were implemented to identify the effect of artichoke bracts and transglutaminase ratio on gluten‐free cookie properties. Artichoke bracts ratio and transglutaminase ratio were independent variables and optimized for responses, namely, moisture, cooking loss, spread ratio, hardness, fracturability, L*, a*, b*, total phenolic content, antioxidant activity, color, texture, flavor, and overall acceptability. In this experimental design, artichoke bracts were included in the range of 0%–30% and transglutaminase enzyme was included in the range of 0%–1%. The model equation proposed for responses included *b*_0_ constant, *b*_i_ linear, *b*_ii_ quadratic, and *b*_ij_ interaction constants; *X* values were independent variables (Equation 1).

(1) $$R=b_{0}+\sum b_{i}X_{i}+\sum b_{\mathrm{ii}}{X_{\mathrm{ii}}}^{2}+\sum b_{\mathrm{ij}}X_{i}X_{j}$$

### 3.2 Artichoke Bracts Powder Production

**FIGURE 1:**
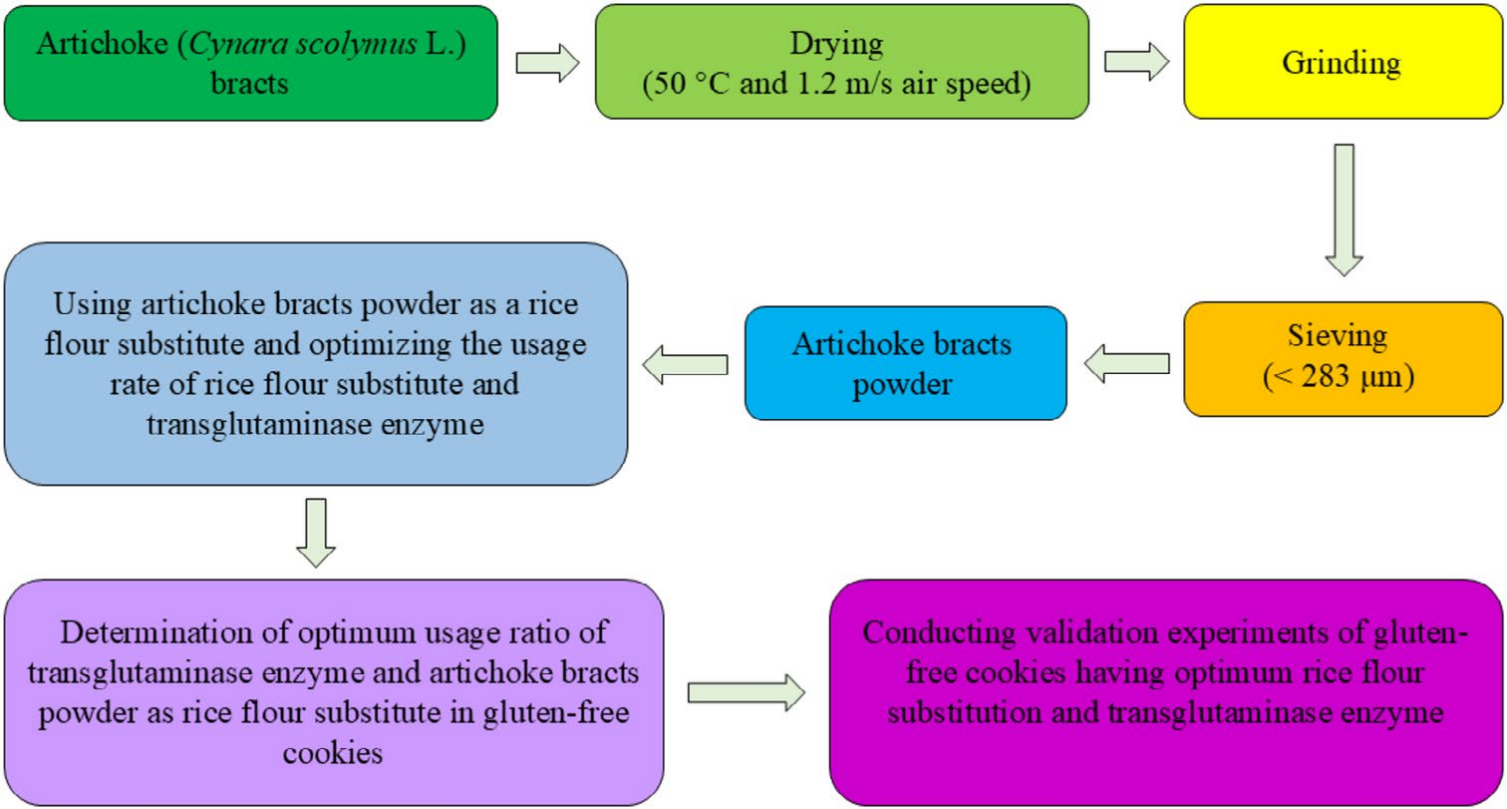
Experimental process.

Artichoke bracts were dried in a laboratory‐type tray dryer (Eksis, Turkey) at 50°C and 1.2 m/s air speed. The drying process was ended when the moisture content of the artichoke bracts dropped below 10% (Lavecchia et al. 2019). The dried artichoke bracts were ground in a blender (Waring, USA) and then passed through a 283 μm mesh sieve. Then sieved artichoke bracts were made ready for use in gluten‐free cookie samples (Figure 1).

### 3.3 Gluten‐Free Cookie Production

The method suggested by Altındağ et al. (2015) was used for gluten‐free cookie production with modification. The formulation of the control gluten‐free cookie comprised 140.00 g rice flour, 60.90 g sugar, 56.00 g margarine, 63.40 g water, 1.40 g skimmed milk powder, 2.10 g baking powder, and 1.75 g salt. Artichoke bracts were substituted for rice flour at a rate of 0%–30%, and transglutaminase enzyme was added to the cookie mixture at a rate of 0%–1% (Table 1). Margarine, sugar, salt, and skimmed milk powder were mixed with a mixer for 2 min (Fakir, Germany). Then, the baking powder and water mixture was added and mixed for 1 min. Finally, rice flour, artichoke bracts powder, and transglutaminase were added, and the dough was manually kneaded for 5 min. The resulting gluten‐free cookie dough was then sheeted to a thickness of 1 cm. The gluten‐free cookies were shaped to a height of 1 cm using a 5 cm diameter cookie cutter. The gluten‐free cookies were baked in an oven at 170°C (Bosch, Türkiye) for a period of 40 min. The baked gluten‐free cookies were left to cool at room temperature for 1 h before being analyzed.

**TABLE 1:** Experimental design, independent variables, and responses obtained from face‐centered central composite design.

Run	Rice flour substitute (%)	Transglutaminase enzyme (%)	Moisture (%)	Cooking loss (%)	Spread ratio	Hardness (N)	Fracturability (mm)	L*	a*	b*
1	0	1	14.57	16.30	5.91	7.32	26.03	85.92	−0.35	22.57
2	15	0.5	14.08	17.41	5.32	23.27	41.99	51.34	4.81	19.63
3	15	0.5	14.12	17.39	5.29	23.75	41.25	51.56	4.73	19.56
4	15	0	13.75	18.35	5.20	25.82	42.76	51.00	4.68	19.73
5	30	0	15.55	16.80	5.11	29.38	45.31	46.26	6.12	18.01
6	0	0	12.98	18.39	5.56	17.74	26.95	83.33	−0.32	21.74
7	15	1	14.38	16.57	5.43	21.57	38.90	52.74	4.78	20.02
8	15	0.5	14.01	17.53	5.32	24.02	41.53	51.92	4.85	19.73
9	30	0.5	15.65	16.03	5.26	28.01	43.87	47.33	6.11	17.79
10	0	0.5	13.71	17.83	5.73	13.50	26.59	83.92	−0.43	21.95
11	30	1	16.02	15.12	5.36	27.19	42.91	48.06	6.25	18.38
12	15	0.5	14.17	17.29	5.31	23.39	41.80	51.27	4.78	19.69
13	15	0.5	14.10	17.49	5.31	23.21	42.03	52.02	4.82	19.12

Run	Rice flour substitute (%)	Transglutaminase enzyme (%)	Total phenolic compound (mg GAE/g)	Antioxidant activity (μmol TE/g)	Color	Texture	Flavor	Overall acceptability
1	0	1	0.41	0.40	6.03	6.26	6.23	6.69
2	15	0.5	0.51	2.42	6.67	6.72	7.94	8.44
3	15	0.5	0.53	2.53	6.59	6.82	7.85	8.29
4	15	0	0.47	2.40	6.73	6.69	7.54	8.17
5	30	0	0.72	3.51	7.75	6.23	8.13	8.61
6	0	0	0.18	0.39	6.12	6.12	6.05	6.24
7	15	1	0.57	2.43	6.50	6.96	7.67	8.53
8	15	0.5	0.49	2.45	6.71	6.64	7.92	8.34
9	30	0.5	0.89	3.53	7.82	6.49	8.18	8.76
10	0	0.5	0.30	0.37	6.09	6.17	6.11	6.43
11	30	1	0.86	3.59	7.55	6.54	8.32	8.82
12	15	0.5	0.56	2.39	6.62	6.89	7.63	8.23
13	15	0.5	0.52	2.42	6.70	6.76	7.87	8.35

### 3.4 Methods

Moisture, cooking loss, spread ratio, texture, color, total phenolic compounds, antioxidant activity analysis, and sensory evaluation were performed on gluten‐free cookies produced according to the experimental design. In addition to these analyses, ash, fat, protein, and dietary fiber analyses were also performed on the optimum gluten‐free cookie and control cookie.

### 3.5 Composition Analysis

The moisture analysis of gluten‐free cookies was implemented using AACC 44‐15 method (AACC 2000). Ash, fat, protein, and dietary fiber analysis were performed according to AOAC 923.03, 945.16, 968.06, and 991.41 (AOAC 2007).

### 3.6 Cooking Loss

The weight of five cookie doughs before baking and the weight of five cookies that were baked and cooled for 1 h were weighed to calculate cooking loss. The difference between the weight of the cookie dough before baking and the weight of the cookies after baking was divided by the weight of the cookie dough to calculate the % cooking loss (Korese et al. 2021).

### 3.7 Spread Ratio

The spread ratio of cookies was determined by AACC method 10‐50D (AACC 2000). Firstly, the diameter and height of gluten‐free cookies were measured using a vernier caliper to calculate the spread ratio. Four gluten‐free cookies were placed on each other and their height was measured. Cookies were replaced in random order and the height was measured again, then the average cookie height was obtained. The diameter of four cookies was measured again after rotating each cookie to 90° and the average cookie diameter was determined. The spread ratio of cookies was calculated from the ratio of the average diameter and the average height of cookies.

### 3.8 Texture Analysis

The texture analysis of gluten‐free cookies was performed by a texture analyzer (TA.XT plus, Stable Micro Systems Ltd., Godalming, UK). A three point bending probe (HDP/3PB) equipped with a 5 kg load cell was used in texture analysis. The hardness and fracturability properties of cookies were determined by three‐point bending test. Pretest speed and test speed were determined as 1.0 and 3.0 mm/s, respectively, and the distance was set to 5.0 mm (Hwang et al. 2016).

### 3.9 Color Analysis

For color analysis, measurements were performed from the top surface of the cookies using a colorimeter (Konica Minolta, CR‐300, Japan). The measured color values were expressed as L* (0: black, 100: white), a* (+*a*: red, −*a*: green) and b* (+*b*: yellow, −*b*: blue).

### 3.10 Total Phenolic Compounds and Antioxidant Activity

To obtain extract to be used for total phenolic compound and antioxidant activity analysis, 2.5 g of ground gluten‐free cookie was extracted with 20 mL of 80% (V/V) methanol at 50°C in a shaking water bath. The extraction process was continued for 90 min at a stirring speed of 150 rpm. At the end of the extraction period, the extract was centrifugated at 4500 rpm for 5 min. Then, the supernatant was filtered through filter paper and its volume was diluted to 25 mL (Heimler et al. 2006).

Total phenolic compounds of gluten‐free cookies were determined by the Folin–Ciocalteu method. Gallic acid solutions were prepared at appropriate concentrations to obtain a standard curve. The total phenolic compounds were calculated as mg gallic acid equivalent (GAE)/g sample with the help of the standard curve (Dadalı 2023a).

DPPH method was used to determine antioxidant activity. In the analysis, 1950 μL of 100 μM DPPH was added to 25, 50, and 75 μL of gluten‐free cookie extracts. They were incubated for 20 min at room temperature, and absorbance values were determined at 517 nm with a spectrophotometer (Agilent, Cary 60, USA). A total of 1950 μL of 100 μM DPPH solution was added to 50 μL of Trolox standard solutions at appropriate concentrations. After 20 min, the absorbance values of the standards were measured at 517 nm by spectrophotometer. Antioxidant activity of gluten‐free cookies was expressed as μmol trolox equivalent (TE)/g sample using percentage inhibition values and trolox standards (Dadalı 2024).

### 3.11 Sensory Evaluation

Sensory evaluation of gluten‐free cookies was carried out with the participation of 67 panelists aged between 17 and 59. Panelists evaluated the samples in individual sensory analysis booths. Gluten‐free cookies were served on a white plate at room temperature. Drinking water was available as a palate cleanser between the gluten‐free cookies. Gluten‐free cookie samples were presented to the panelists with 3‐digit randomly selected codes. Color, texture, flavor, and overall acceptability properties of gluten‐free cookies were evaluated. A 9‐point hedonic (1: dislike extremely, 9: like very much) scale was used in sensory evaluation.

### 3.12 Statistical Analysis

The modeling and optimization were performed by Design‐Expert 13.0 (Design Expert 2020). Statistical evaluation of control and optimized gluten‐free cookie properties was carried out by ANOVA using the 20.0 package program (SPSS 2012). Duncan's multiple range test was utilized to define statistically significant differences (*α* = 0.05).

## 4 Results and Discussion

This study aimed to investigate the potential use of artichoke bracts as a substitute for rice flour in the formulation of gluten‐free cookies, with a particular focus on optimizing the substitution level and the concentration of transglutaminase enzyme. The rice flour substitute and transglutaminase were defined as independent variables and evaluated within the range of 0%–30% and 0%–1%, respectively.

### 4.1 Model Descriptions

**FIGURE 2:**
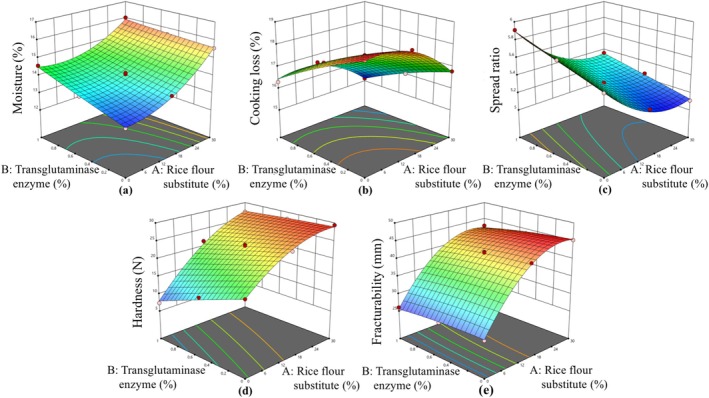
The calculated effect of rice flour substitute and transglutaminase on moisture (a), cooking loss (b), spread ratio (c), hardness (d), and fracturability (e).

**FIGURE 3:**
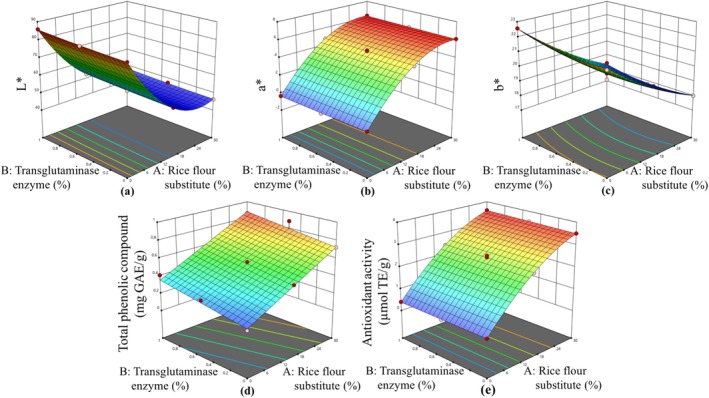
The calculated effect of rice flour substitute and transglutaminase on L* (a), a* (b), b* (c), total phenolic compound (d), and antioxidant activity (e).

**FIGURE 4:**
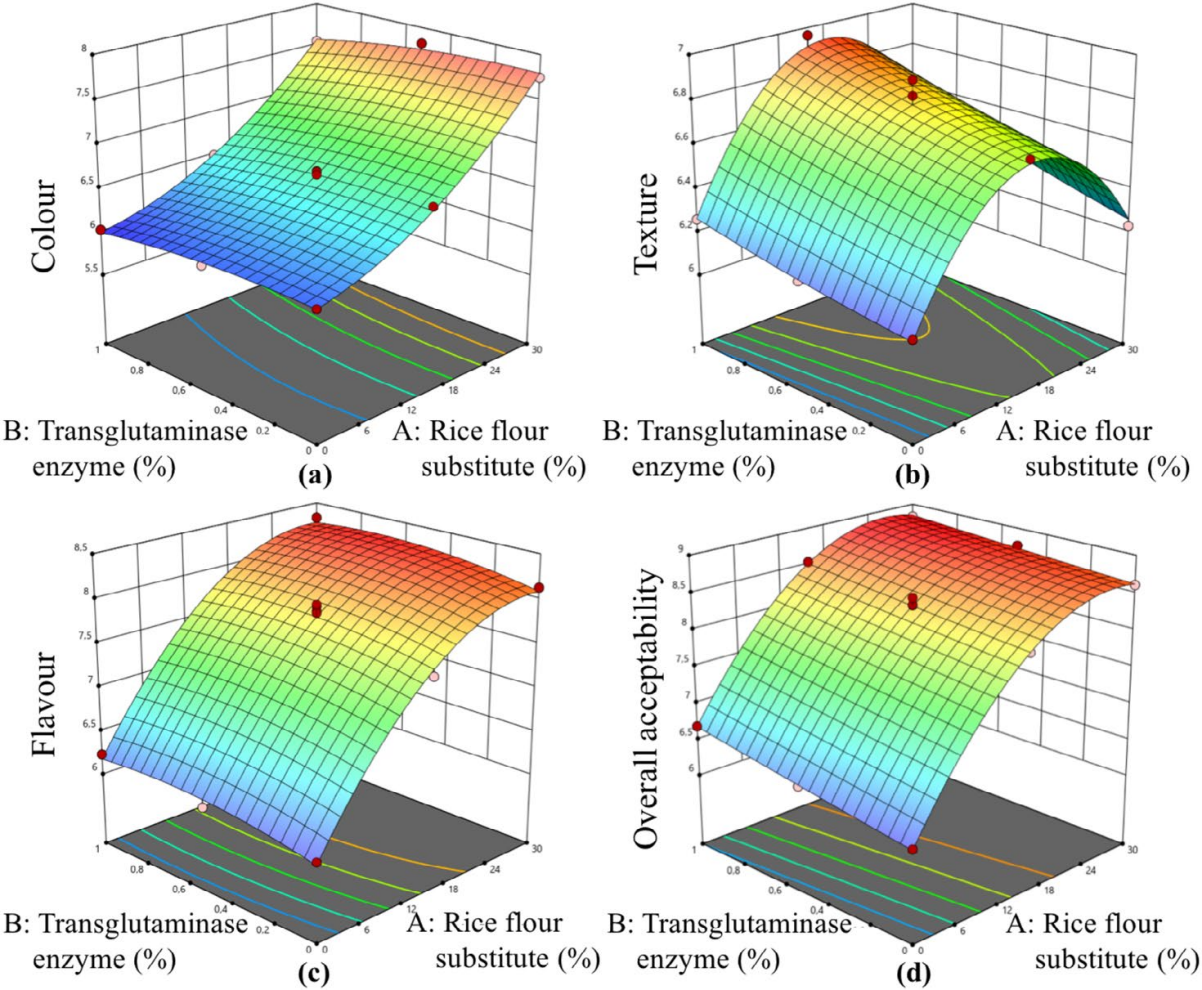
The calculated effect of rice flour substitute and transglutaminase on color (a), texture (b), flavor (c), and overall acceptability (d).

The effect of rice flour substitute and transglutaminase ratio on gluten‐free cookies was investigated by response surface methodology with FCCCD having 13 runs. The statistical significance of model terms was determined by ANOVA (Table 2). All models were statistically significant and were utilized to determine the effect of rice flour substitute and transglutaminase on responses (*p* < 0.01). According to ANOVA results, the lack of fit values was insignificant (*p* > 0.05), which shows the adequacy of fitted models. Responses, namely, moisture content, cooking loss, spread ratio, hardness, fracturability, L*, a*, b*, total phenolic content, antioxidant activity, color, texture, flavor, and overall acceptability were explained with a quadratic model, while total phenolic content was explained with a linear model. The regression coefficient (*R* ^2^) and adjusted regression coefficient (Adj‐*R* ^2^) values were determined for models (Table 3). All *R* ^2^ values were higher than 0.9497, which showed that the obtained data were in agreement with fitted models. The counterplots of the predicted models for responses are shown in Figures 2, 3, 4.

**TABLE 2:** ANOVA results indicating linear, quadratic, interaction effects, and the lack of fit of the responses.

Source	Sum of squares	df	Mean square	*F*‐value	*p*	Source	Sum of squares	df	Mean square	*F*‐value	*p*
**R_1_: Moisture**						**R_2_: Cooking loss**					
Model	8.88	5.00	1.78	156.17	0.00	Model	10.21	5.00	2.04	88.63	0.00
*X*_1_	5.93	1.00	5.93	522.04	0.00	*X*_1_	3.49	1.00	3.49	151.62	0.00
*X*_2_	1.21	1.00	1.21	106.86	0.00	*X*_2_	5.11	1.00	5.11	221.89	0.00
*X*_1_ *X*_2_	0.31	1.00	0.31	27.47	0.00	*X*_1_ *X*_2_	0.04	1.00	0.04	1.87	0.21
*X*_1_^2^	1.16	1.00	1.16	102.24	0.00	*X*_1_^2^	1.14	1.00	1.14	49.56	0.00
*X*_2_^2^	0.00	1.00	0.00	0.26	0.63	*X*_2_^2^	0.04	1.00	0.04	1.60	0.25
Lack of Fit	0.07	3.00	0.02	6.40	0.05	Lack of Fit	0.13	3.00	0.04	4.84	0.08
**R_3_: Spread ratio**						**R_4_: Hardness**					
Model	0.58	5.00	0.12	301.29	0.00	Model	447.40	5.00	89.48	242.31	0.00
*X*_1_	0.36	1.00	0.36	937.71	0.00	*X*_1_	352.97	1.00	352.97	955.83	0.00
*X*_2_	0.11	1.00	0.11	298.95	0.00	*X*_2_	47.38	1.00	47.38	128.29	0.00
*X*_1_ *X*_2_	0.00	1.00	0.00	6.51	0.04	*X*_1_ *X*_2_	16.93	1.00	16.93	45.85	0.00
*X*_1_^2^	0.09	1.00	0.09	227.23	0.00	*X*_1_^2^	25.21	1.00	25.21	68.27	0.00
*X*_2_^2^	0.00	1.00	0.00	0.04	0.85	*X*_2_^2^	0.02	1.00	0.02	0.05	0.83
Lack of Fit	0.00	3.00	0.00	4.64	0.09	Lack of Fit	2.11	3.00	0.70	5.88	0.06
**R_5_: Fracturability**						R_6_: L*					
Model	593.12	5.00	118.62	305.30	0.00	Model	2722.14	5.00	544.43	4876.69	0.00
*X*_1_	459.73	1.00	459.73	1183.18	0.00	*X*_1_	2072.41	1.00	2072.41	18,563.56	0.00
*X*_2_	8.59	1.00	8.59	22.11	0.00	*X*_2_	6.26	1.00	6.26	56.10	0.00
*X*_1_ *X*_2_	0.55	1.00	0.55	1.41	0.27	*X*_1_ *X*_2_	0.16	1.00	0.16	1.40	0.27
*X*_1_^2^	100.31	1.00	100.31	258.17	0.00	*X*_1_^2^	542.14	1.00	542.14	4856.21	0.00
*X*_2_^2^	0.50	1.00	0.50	1.29	0.29	*X*_2_^2^	0.18	1.00	0.18	1.65	0.24
Lack of Fit	2.29	3.00	0.76	7.05	0.05	Lack of Fit	0.33	3.00	0.11	0.95	0.50
R_7_: a*						R_8_: b*					
Model	75.37	5.00	15.07	4316.02	0.00	Model	25.61	5.00	5.12	124.76	0.00
*X*_1_	63.91	1.00	63.91	18299.06	0.00	*X*_1_	24.30	1.00	24.30	591.84	0.00
*X*_2_	0.01	1.00	0.01	1.87	0.21	*X*_2_	0.37	1.00	0.37	9.06	0.02
*X*_1_ *X*_2_	0.01	1.00	0.01	1.79	0.22	*X*_1_ *X*_2_	0.05	1.00	0.05	1.28	0.30
*X*_1_^2^	9.81	1.00	9.81	2809.74	0.00	*X*_1_^2^	0.27	1.00	0.27	6.61	0.04
*X*_2_^2^	0.00	1.00	0.00	0.04	0.85	*X*_2_^2^	0.28	1.00	0.28	6.78	0.04
Lack of Fit	0.02	3.00	0.01	2.64	0.19	Lack of Fit	0.04	3.00	0.01	0.25	0.86
**R_9_: Total phenolic compound**						**R_10_: Antioxidant activity**					
Model	0.45	2.00	0.23	120.68	0.00	Model	15.64	5.00	3.13	1619.85	0.00
*X*_1_	0.42	1.00	0.42	222.03	0.00	*X*_1_	14.92	1.00	14.92	7727.71	0.00
*X*_2_	0.04	1.00	0.04	19.33	0.00	*X*_2_	0.00	1.00	0.00	1.39	0.28
Lack of Fit	0.02	6.00	0.00	3.81	0.11	*X*_1_ *X*_2_	0.00	1.00	0.00	0.58	0.47
						*X*_1_^2^	0.61	1.00	0.61	315.32	0.00
						*X*_2_^2^	0.00	1.00	0.00	0.00	0.97
						Lack of Fit	0.00	3.00	0.00	0.22	0.88
**R_11_: Color**						**Response_12_: Texture**					
Model	4.22	5.00	0.84	260.18	0.00	Model	0.93	5.00	0.19	26.43	0.00
*X*_1_	3.96	1.00	3.96	1221.08	0.00	*X*_1_	0.08	1.00	0.08	11.92	0.01
*X*_2_	0.05	1.00	0.05	14.17	0.01	*X*_2_	0.09	1.00	0.09	12.23	0.01
*X*_1_ *X*_2_	0.00	1.00	0.00	1.02	0.35	*X*_1_ *X*_2_	0.01	1.00	0.01	1.02	0.35
*X*_1_^2^	0.20	1.00	0.20	63.23	0.00	*X*_1_^2^	0.66	1.00	0.66	93.37	0.00
*X*_2_^2^	0.01	1.00	0.01	3.78	0.09	*X*_2_^2^	0.00	1.00	0.00	0.07	0.79
Lack of Fit	0.01	3.00	0.00	1.52	0.34	Lack of Fit	0.01	3.00	0.00	0.48	0.71
**R_13_: Flavor**						**Response_14_: Overall acceptability**					
Model	7.74	5.00	1.55	93.76	0.00	Model	9.75	5.00	1.95	504.42	0.00
*X*_1_	6.49	1.00	6.49	392.87	0.00	*X*_1_	7.77	1.00	7.77	2010.90	0.00
*X*_2_	0.04	1.00	0.04	2.52	0.16	*X*_2_	0.17	1.00	0.17	44.85	0.00
*X*_1_ *X*_2_	0.00	1.00	0.00	0.00	0.97	*X*_1_ *X*_2_	0.01	1.00	0.01	3.72	0.09
*X*_1_^2^	0.88	1.00	0.88	53.36	0.00	*X*_1_^2^	1.54	1.00	1.54	398.69	0.00
*X*_2_^2^	0.03	1.00	0.03	1.84	0.22	*X*_2_^2^	0.00	1.00	0.00	0.04	0.84
Lack of Fit	0.05	3.00	0.02	1.14	0.43	Lack of Fit	0.00	3.00	0.00	0.16	0.92

Abbreviations: *X*_1_, rice flour substitute (%); *X*_2_, transglutaminase enzyme (%).

**TABLE 3:** Model equations and parameters of responses.

Response	Equation	*R* ^2^	Adj‐*R* ^2^	C.V. (%)	Adeq precision
Moisture	14.08 + 0.9945 *X*_1_ − 0.4500 *X*_2_ − 0.2794 *X*_1_ *X*_2_ − 0.6487 *X*_1_^2^ − 0.0325 *X*_2_^2^	0.9911	0.9848	0.7409	39.8840
Cooking loss	17.46 − 0.7631 *X*_1_ − 0.9231 *X*_2_ + 1038 *X*_1_ *X*_2_ − 0.6430 *X*_1_^2^ − 0.1155 *X*_2_^2^	0.9845	0.9735	0.8869	32.7017
Spread ratio	5.31 − 0.2450 *X*_1_ + 0.1383 *X*_2_ − 0.0250*X*_1_ *X*_2_ + 0.1778 *X*_1_^2^ − 0.0022 *X*_2_^2^	0.9954	0.9921	0.3634	57.5832
Hardness	23.6000 − 7.6700 *X*_1_ − 2.8100 *X*_2_ + 2.0600 *X*_1_ *X*_2_ − 3.0200 *X*_1_^2^ − 0.0814 *X*_2_^2^	0.9943	0.9902	2.7400	50.7700
Fracturability	41.5900 + 8.7500 *X*_1_ − 1.2000 *X*_2_ − 0.3700*X*_1_ *X*_2_ − 6.0300 *X*_1_^2^ − 0.4266 *X*_2_^2^	0.9954	0.9922	1.6100	46.992
L*	51.6200 − 18.5900 *X*_1_ + 1.0200 *X*_2_ − 0.1980*X*_1_ *X*_2_ + 14.01 *X*_1_^2^ + 0.2584 *X*_2_^2^	0.9997	0.9995	0.5740	172.7512
a*	4.7800 + 3.26*X*_1_ + 0.0330 *X*_2_ + 0.0395*X*_1_ *X*_2_ − 1.88 *X*_1_^2^ + 0.0071 *X*_2_^2^	0.9997	0.9994	1.5100	164.5610
b*	19.55–2.0100 *X*_1_ + 0.2490 *X*_2_ − 0.1145*X*_1_ *X*_2_ + 0.3136 *X*_1_^2^ + 0.3176 *X*_2_^2^	0.9889	0.9810	1.0200	34.1859
Total phenolic content	0.5396 + 0.2630 *X*_1_ + 0.0776 *X*_2_	0.9602	0.9523	8.0100	32.7991
Antioxidant activity	2.43 + 1.58 *X*_1_ + 0.212 *X*_2_ + 0.0168 *X*_1_ *X*_2_ − 0.4696 *X*_1_^2^ − 0.0011 *X*_2_^2^	0.9991	0.9985	1.9800	107.0709
Color	6.66 + 0.8122 *X*_1_ − 0.0875 *X*_2_ − 0.0287 *X*_1_ *X*_2_ + 0.2724 *X*_1_^2^ − 0.066 *X*_2_^2^	0.9946	0.9908	0.8423	46.5221
Texture	6.78 + 0.1185 *X*_1_ + 0.1200 *X*_2_ − 0.0425 *X*_1_ *X*_2_ − 0.4887 *X*_1_^2^ + 0.0138 *X*_2_^2^	0.9497	0.9138	1.28	14.0917
Flavor	7.80 + 1.04 *X*_1_ + 0.0833 *X*_2_ + 0.0025 *X*_1_ *X*_2_ − 0.5649 *X*_1_^2^ − 0.1049 *X*_2_^2^	0.9853	0.9748	1.71	25.9489
Overall acceptability	8.33 + 1.14 *X*_1_ + 0.1700 *X*_2_ − 0.0600 *X*_1_ *X*_2_ − 0.7471 *X*_1_^2^ + 0.0079 *X*_2_^2^	0.9972	0.9953	0.7780	61.9433

Abbreviations: *X*_1_, rice flour substitute (%); *X*_2_, transglutaminase enzyme (%).

### 4.2 Physical Properties

The rice flour substitute exhibited a linear and quadratic influence on the moisture content of gluten‐free cookies. While the transglutaminase demonstrated a linear response on moisture content, an interaction effect between the rice flour substitute and the transglutaminase enzyme was also observed (*p* < 0.05). As the rice flour substitute used in gluten‐free cookies increased, an increase in moisture content was observed. Similarly, the use of transglutaminase in gluten‐free cookies increased the moisture content. It was demonstrated that products with high fiber content possess a high water‐holding capacity. Consequently, when these substitutes were utilized in cookies, a decrease in baking loss and an increase in moisture content were observed. The moisture content of the gluten‐free cookie devoid of rice flour substitute and transglutaminase (Exp. 6) was 12.98%, whereas the moisture content of the gluten‐free cookie containing 30% rice flour substitute and 1% transglutaminase was determined to be 16.02% (Table 1). An inverse relationship was observed between baking loss and moisture content, with the product exhibiting the lowest moisture content demonstrating the highest baking loss. The findings of this study are consistent with the results of Naknaen et al. (2016) and Gagneten et al. (2023), who detected an increase in moisture content of cookies having watermelon rind as a flour substitute and moisture content of gluten‐free cookies containing black currant waste. In addition, Costantini et al. (2024) found that the moisture content of cookies with hazelnut skins increased with hazelnut skins concentration. In the study conducted by Altındağ et al. (2015), it was demonstrated that the moisture content of cookies increased as a result of the use of transglutaminase. The mechanism by which the moisture content is increased by the transglutaminase enzyme involves modification of the transglutaminase protein structure and induction of the conversion of glutamine to glutamic acid. Consequently, the water binding capacity of proteins is augmented due to the increase in glutamic acid (Beck et al. 2011).

The spread ratio of gluten‐free cookies exhibited a range from 5.11 to 5.91 (Table 1). The rice flour substitution demonstrated a linear and quadratic response to spread ratio (*p* < 0.05). The transglutaminase enzyme exerted a linear influence on the spreading ratio, and an interaction effect was observed between rice flour substitution and transglutaminase (*p* < 0.05). It was demonstrated that the spread ratio decreased as the rice flour substitute usage ratio increased. However, the spread ratio increased in accordance with the increase in the transglutaminase usage ratio. In the study conducted by Gruppi et al. (2024), a reduction in the spread ratio of cookies containing bamboo, cocoa, citrus, and chokeberry fiber was observed. In the study where a mixture of buckwheat and rice flour was utilized in the production of gluten‐free cookies, the spread ratio, which was 5.66, increased to 5.90 with the incorporation of transglutaminase into the gluten‐free cookie mixture (Altındağ et al. 2015).

### 4.3 Texture Analysis

This study evaluated the effects of rice flour substitution on textural properties, namely, hardness and fracturability of gluten‐free cookies. The linear and quadratic effects of rice flour substitution, as well as the linear effect of transglutaminase enzyme on hardness were determined to be statistically significant (*p* < 0.05). Furthermore, the interaction effect of rice flour substitution and transglutaminase enzyme on the hardness of gluten‐free cookies was statistically significant (*p* < 0.05). The results showed that the hardness values were affected linearly and quadratically by substituting rice flour, and linearly by transglutaminase enzyme (*p* < 0.05). The incorporation of rice flour substitute in the production of gluten‐free cookies led to an enhancement in hardness, while the utilization of transglutaminase enzyme in gluten‐free cookies resulted in a reduction in hardness. The lowest recorded hardness value (7.32 N) was observed in the sample containing 1% transglutaminase and no rice flour substitution (Exp. 1), while the hardness of the gluten‐free cookie containing 30% rice flour substitution and without transglutaminase (29.38 N) was higher than the other cookies (Exp. 5; Table 1). A similar trend was observed in the fracturability values of gluten‐free cookies, which increased with the use of rice flour substitute and decreased with the use of transglutaminase. The utilization of components characterized by elevated levels of fiber content was demonstrated to result in an increase in the hardness value. The use of buriti endocarp flour having abundant fiber content in gluten‐free cookies resulted in an enhancement of hardness (Becker et al. 2014). In a similar manner, the hardness value of bread increased from 12.10 N to 21.40 N when 10% artichoke bracts were used (Canale et al. 2022). The incorporation of transglutaminase in gluten‐free bread production also led to a reduction in bread hardness. The augmentation in water holding capacity of transglutaminase provides a rationale for the decrease in hardness of gluten‐free biscuits (Dłużewska et al. 2015). Similarly, in a study where cocoa shell was used as a flour substitute at a level of 20% in cookie formulation, an increase in the fracturability of the cookies was observed due to the elevated fiber content (Handojo et al. 2019).

### 4.4 Color Analysis

Linear and quadratic effects of the utilization ratio of rice flour substitution on L* (*p* < 0.05) were identified, as had linear and quadratic effects of the transglutaminase enzyme present in gluten‐free cookies on L* (*p* < 0.05; Table 2). The utilization of rice flour substitute in gluten‐free cookies resulted in a decline in L* values, with the highest L* value recorded as 85.92 (Exp. 1) and the lowest L* value as 46.26 (Exp. 5). Despite alterations in the usage ratio of transglutaminase enzyme in gluten‐free cookies containing equivalent amounts of rice flour substitute, the L* values remained relatively similar. In contrast, transglutaminase did not have an effect on the a* value of gluten‐free cookies (*p* > 0.05), despite the linear and quadratic effects of rice flour substitution on a* (*p* < 0.05). Conversely, the a* values of gluten‐free cookies exhibited an increase with rising rice flour substitute usage. Negative a* values were observed in gluten‐free cookies devoid of rice flour substitute. It was determined that a* values became positive with the incorporation of rice flour substitute in gluten‐free cookies. The findings indicate that both rice flour substitution and the transglutaminase enzyme exert a linear and quadratic influence on the b* value (*p* < 0.05). The b* values exhibited a decrease with increasing usage rates of rice flour substitute. The b* values of gluten‐free cookies containing 0%, 15%, and 30% rice flour substitute were in the range of 21.74–22.57, 19.12–20.02, and 17.79–18.38, respectively (Table 1). The findings of the present study are consistent with the study conducted by Bavaro et al. (2025), in which artichoke bracts were used as a substitute for wheat flour in bread up to a proportion of 15%. As the proportion of artichoke bracts in bread increased, a decrease in L* and b* values and an increase in a* values were detected. In another study that evaluated the color parameters of transglutaminase use in cookies, it was emphasized that L*, a*, and b* values were close to gluten‐free cookies without glutaminase, in accordance with the results of the present study (Altındağ et al. 2015).

### 4.5 Total Phenolic Content and Antioxidant Activity Analysis

The total phenolic content of gluten‐free cookies was found to be subject to a linear effect arising from the substitution of rice flour, as well as the addition of a transglutaminase enzyme (*p* < 0.05). It was determined that only the substitution of rice flour was effective in increasing antioxidant activity, and both linear and quadratic effects of this substitution were observed (*p* < 0.05). The total phenolic content of gluten‐free cookies was found to increase with the incorporation of rice flour substitute. The total phenolic content of the gluten‐free cookie without artichoke bracts and transglutaminase enzyme (Exp. 6) was 0.18 mg GAE/g, which increased to 0.89 mg GAE/g in the gluten‐free cookie where rice flour substitute was 30% (Exp. 9). The antioxidant activity values of gluten‐free cookies ranged from 0.37 to 3.59 μmol TE/g, and the use of 30% rice flour substitute in gluten‐free cookies resulted in an approximate 10 times increase in antioxidant activity values. A similar relationship was observed between antioxidant activity and total phenolic content. The use of rice flour substitute in gluten‐free cookies was found to be associated with an increase in antioxidant activity. In a previous study, the amount of artichoke bracts used in bread was limited to 6%, and the total phenolic substance was determined to be 0.39 mg GAE/g and the antioxidant activity was 1.50 μmol TE/g (Cannas et al. 2024). In another study, the addition of 7% artichoke bracts to gluten‐free bread resulted in a total phenolic substance content of 1.36 mg GAE/100 g, which was lower than the values reported in this study (Proetto et al. 2024). The elevated total phenolic content and antioxidant activity values determined in this study can be attributed to the increased usage ratio of artichoke bracts. In a separate study, in which artichoke bracts were utilized as a 40% wheat flour substitute in cake, the antioxidant activity value was determined to be 3.12 μmol TE/g (Dadalı 2023a). This result was comparable to the antioxidant activity values (3.51–3.59 μmol TE/g) of gluten‐free cookies, in which a 30% rice flour substitute was incorporated.

### 4.6 Sensory Evaluation

The sensory properties, namely, color and texture, were found to be affected by rice flour substitution in a linear and a quadratic manner (*p* < 0.05). Additionally, a quadratic effect of rice flour substitution was observed (*p* < 0.05; Table 2). The use of artichoke bracts as a substitute for rice flour exerted a linear and quadratic influence on the sensory characteristics of gluten‐free cookies (*p* < 0.05). The overall acceptability of gluten‐free cookies was determined to be subject to a linear and a quadratic change in dependence on the utilization of rice flour substitution. The analysis further reveals a linear effect of the transglutaminase enzyme (*p* < 0.05). The evaluation of the sensory properties of gluten‐free cookies was conducted, encompassing parameters such as color, texture, flavor, and overall acceptability. The color properties of the cookies were found to be positively influenced by rice flour substitute. A positive relation was observed between the incorporation of rice flour substitute and an increase in color scores. The highest color score, 7.82, was observed in the gluten‐free cookie containing 30% rice flour substitute and 0.5% transglutaminase (Exp. 9), which was significantly higher than that of other gluten‐free cookies. Furthermore, an enhancement in the use of rice flour substitute from 0% to 15% in gluten‐free cookies resulted in a favorable change in texture. However, gluten‐free cookies with 30% rice flour substitute exhibited lower texture scores (6.23–6.54) compared to those with 15% rice flour substitute (6.64–6.96; Table 1). Moreover, the addition of a transglutaminase enzyme had a favorable impact on the texture of gluten‐free cookies. The incorporation of rice flour substitute in the formulation of gluten‐free cookies was demonstrated to enhance flavor. The overall acceptability results indicated that gluten‐free cookies with 15% and 30% rice flour substitution were more favored than gluten‐free cookies without artichoke bracts. The study demonstrated that the overall acceptability of gluten‐free cookies increased with the use of transglutaminase.

### 4.7 Optimization and Model Verifications

The numerical optimization of the rice flour substitute and transglutaminase ratio was implemented using the desirability function method. The resultant optimization process yielded the determination of the optimal rice flour substitution rate and transglutaminase ratio as 18.65% and 0.99%, respectively. Five validation experiments were conducted for gluten‐free cookies comprising the optimum rice flour substitution and transglutaminase ratio. The validation experiment results and the data estimated from the model are presented in Table 4. The findings revealed that there was no statistically significant difference between the response data obtained from the model and the experimental response data (*p* < 0.05).

**TABLE 4:** Model predicted responses and experimental results.

Responses	Predicted value	Experimental value^a^	Mean standard error	Difference	Error (%)^b^
Moisture (g/100 g)	14.77	14.98 ± 0.11	0.09	0.21	1.40
Cooking loss (%)	16.23	15.97 ± 0.15	0.13	−0.26	1.64
Spread ratio	5.39	5.36 ± 0.02	0.02	−0.04	0.69
Hardness (N)	22.90	23.38 ± 0.61	0.51	0.49	2.08
Fracturability (mm)	41.65	42.21 ± 0.62	0.52	0.56	1.32
L*	49.16	49.66 ± 0.33	0.28	0.51	1.02
a*	5.51	5.42 ± 0.06	0.05	−0.09	1.72
b*	19.62	19.97 ± 0.20	0.17	0.35	1.76
Total phenolic compound (mg GAE/g)	0.68	0.74 ± 0.04	0.03	0.06	7.92
Antioxidant activity (μmol TE/g)	2.81	2.89 ± 0.04	0.04	0.08	2.61
Color	6.72	6.81 ± 0.06	0.05	0.09	1.39
Texture	6.92	6.81 ± 0.08	0.07	−0.12	1.70
Flavor	8.00	7.89 ± 0.13	0.11	−0.11	1.42
Overall acceptability	8.73	8.78 ± 0.06	0.05	0.05	0.62

^a^ The analysis results were given as arithmetic mean ± SD.

^b^ % Error = (|*y*_exp_ − *y*_pre_|/*y*_exp_) × 100.

### 4.8 Characterization of Control and Optimized Gluten‐Free Cookies

The composition analyses of the optimum gluten‐free cookie, containing the optimum transglutaminase enzyme and artichoke bracts as a substitute for rice flour, are presented in Table 5. A statistically significant increase in moisture content was observed in the optimum gluten‐free cookie in comparison to the control gluten‐free cookie (*p* < 0.05). This phenomenon can be attributed to the elevated water‐retention capabilities of artichoke bracts and the enhanced water‐retention capacity of the transglutaminase enzyme (Beck et al. 2011; Dadalı 2023a). Additionally, a statistically significant increase in ash content was observed in the optimum gluten‐free cookie compared to the control gluten‐free cookie (*p* < 0.05). A similar outcome was observed in a separate study, where the incorporation of artichoke bracts in bread resulted in an increase in ash content (Cannas et al. 2024). However, a statistically insignificant difference was observed between the fat and protein results (*p* > 0.05). In a previous study, the protein content of gluten‐free cookies was determined to be 3.39 g/100 g, which was consistent with the protein content determined in this study (3.73 and 3.75 g/100 g). The study conducted by Rai et al. (2014) found that the fat content of gluten‐free cookies ranged from 15.45 to 19.17 g/100 g, which is similar to the fat results in this study. The total dietary fiber content exhibited an increase in the optimum gluten‐free cookie, attributable to the incorporation of artichoke bracts in contrast to the control gluten‐free cookie (*p* < 0.05). According to the food classification system of the Food and Agriculture Organization of the United Nations (FAO) that prioritizes dietary fiber content, the optimum gluten‐free cookie can be designated as a high‐fiber food, as its dietary fiber content exceeds 6 g/100 g (FAO 2025).

**TABLE 5:** Composition of control and optimum gluten‐free cookie*.

	Control gluten‐free cookie	Optimum gluten‐free cookie
Moisture (g/100 g)	12.98 ± 0.09^a^	14.98 ± 0.11^b^
Ash (g/100 g)	1.92 ± 0.12^a^	2.39 ± 0.08^b^
Fat (g/100 g)	14.96 ± 0.35^a^	14.74 ± 0.16^a^
Protein (g/100 g)	3.73 ± 0.07^a^	3.75 ± 0.17^a^
Total dietary fiber (g/100 g)	2.28 ± 0.06^a^	7.97 ± 0.18^b^

*Note:* Different letters indicate statistical differences within row (*p* < 0.05).

* The analysis results were given as arithmetic mean ± SD.

## 5 Conclusion

Artichoke waste constitutes a significant by‐product with the potential to be utilized within the food industry. Artichoke bracts from these byproducts were utilized as a substitute for rice flour in gluten‐free cookies, thereby promoting sustainable food production and contributing to human health. In this study, artichoke bracts were used as a rice flour substitute in gluten‐free cookies, and the rice flour substitute ratio and transglutaminase ratio were optimized. The study's findings indicated that the optimum ratio of rice flour substitute was determined to be 18.65%, and the transglutaminase enzyme ratio was 0.99%. The analysis revealed that the optimum gluten‐free cookie exhibited increased moisture content, hardness, and brittleness values compared to the control cookie. Conversely, the cooking loss and spreading rate exhibited a decline. Notably, the antioxidant activity value and total phenolic compound content of the optimum gluten‐free cookie increased, and its sensory properties improved. Notably, despite the reduced dietary fiber content characteristic of gluten‐free products, the optimized gluten‐free cookie can be regarded as a high‐fiber food. This study addressed a critical concern in the food industry, namely the disposal of artichoke bracts, and successfully developed a new, value‐added gluten‐free product. This new gluten‐free cookie is poised to enhance the nutritional content of gluten‐free diets. Subsequent studies can investigate the potential of artichoke bracts in the development of novel and healthy food products.

## Author Contributions

**Ceyda Dadalı:** conceptualization (equal), formal analysis (equal), investigation (equal), methodology (equal), supervision (equal), writing – original draft (equal), writing – review and editing (equal). **Yağmur Özcan:** formal analysis (equal), investigation (equal), methodology (equal), writing – original draft (equal), writing – review and editing (equal). **İrem Cansu Ensari:** formal analysis (equal), investigation (equal), methodology (equal).

## Conflicts of Interest

The authors declare no conflicts of interest.

## Data Availability

The data that support the findings of this study are available from the corresponding author upon reasonable request.
